# Digging the New York City Skyline: Soil Fungal Communities in Green Roofs and City Parks

**DOI:** 10.1371/journal.pone.0058020

**Published:** 2013-03-01

**Authors:** Krista L. McGuire, Sara G. Payne, Matthew I. Palmer, Caitlyn M. Gillikin, Dominique Keefe, Su Jin Kim, Seren M. Gedallovich, Julia Discenza, Ramya Rangamannar, Jennifer A. Koshner, Audrey L. Massmann, Giulia Orazi, Adam Essene, Jonathan W. Leff, Noah Fierer

**Affiliations:** 1 Department of Biology, Barnard College of Columbia University, New York, New York, United States of America; 2 Department of Ecology, Evolution and Environmental Biology, Columbia University, New York, New York, United States of America; 3 Department of Environmental Science, Barnard College of Columbia University, New York, New York, United States of America; 4 Department of Biology, Fordham University, Bronx, New York, United States of America; 5 Cooperative Institute for Research in Environmental Sciences, University of Colorado, Boulder, Colorado, United States of America; 6 Department of Ecology and Evolutionary Biology, University of Colorado, Boulder, Colorado, United States of America; Argonne National Laboratory, United States of America

## Abstract

In urban environments, green roofs provide a number of benefits, including decreased urban heat island effects and reduced energy costs for buildings. However, little research has been done on the non-plant biota associated with green roofs, which likely affect their functionality. For the current study, we evaluated whether or not green roofs planted with two native plant communities in New York City functioned as habitats for soil fungal communities, and compared fungal communities in green roof growing media to soil microbial composition in five city parks, including Central Park and the High Line. Ten replicate roofs were sampled one year after planting; three of these roofs were more intensively sampled and compared to nearby city parks. Using Illumina sequencing of the fungal ITS region we found that green roofs supported a diverse fungal community, with numerous taxa belonging to fungal groups capable of surviving in disturbed and polluted habitats. Across roofs, there was significant biogeographical clustering of fungal communities, indicating that community assembly of roof microbes across the greater New York City area is locally variable. Green roof fungal communities were compositionally distinct from city parks and only 54% of the green roof taxa were also found in the park soils. Phospholipid fatty acid analysis revealed that park soils had greater microbial biomass and higher bacterial to fungal ratios than green roof substrates. City park soils were also more enriched with heavy metals, had lower pH, and lower quantities of total bases (Ca, K, and Mg) compared to green roof substrates. While fungal communities were compositionally distinct across green roofs, they did not differentiate by plant community. Together, these results suggest that fungi living in the growing medium of green roofs may be an underestimated component of these biotic systems functioning to support some of the valued ecological services of green roofs.

## Introduction

Green roofs have become increasingly popular in urban sustainability initiatives, as they provide a number of ecosystem services that mitigate the effects of urbanization such as decreased storm water runoff, enhanced building energy-use efficiency, and reduced urban heat island effects [1]–[3]. An additional benefit of green roofs that has not been fully explored is the potential reservoir of habitats for biota residing in or migrating across the city [4]. Like city parks and other urban green spaces, green roofs provide vegetated islands that birds, insects, and other airborne organisms may make use of in the urban matrix [5]–[8]. However, the historical focus of green roof research has been on infrastructure and engineering, so the role of green roofs as biodiversity reservoirs has only recently been emphasized [5]. Understanding how biodiversity is assembled and maintained will be useful for managing green roof systems to maximize their provision of ecosystem services while simultaneously minimizing external inputs and roof maintenance. In addition to their practical aspects, green roofs can also function as ideal experimental systems for asking ecological questions about community assembly and habitat fragmentation.

The community composition of the vegetation planted on green roofs may have a major impact on their associated biodiversity. Most green roofs in North America are planted with non-native species of *Sedum* (Crassulaceae), which are succulent, perennial ground plants tolerant to the extreme conditions found on rooftops [9], [10]. However, if one of the aims of installing a green roof is to maintain local biodiversity, then non-native *Sedum* may not be the optimal vegetation choice. One of the challenges of installing native plants on green roofs is that they must be able to withstand the harsh rooftop environments and demonstrate performance that equals or surpasses monocultures of *Sedum* in terms of the additional ecosystem services they provide. A recent study in Nova Scotia found that mixtures of *Sedum* and different life forms of native plants, such as grasses and forbs, displayed optimum performance in terms of building temperature reduction and water capture [11]. However the associated non-plant biodiversity of these communities was not assessed. Positive links between biodiversity and ecosystem function have long been recognized in plant community ecology, but since engineers and architects have led most green roof initiatives [12], attention to the particular plant community installed on the roofs has not been a focus. In fact, a recent review of the green roof literature identified only five studies that had specifically manipulated plant diversity in green roof communities [13]. From an ecological perspective, if green roofs are to function as effective biodiversity reservoirs, then the particular assemblage of plants on the roof may have a major impact on which non-plant taxa are attracted to and can utilize the habitat in different regions.

Fungi residing in the growing media are one of the integral components of the green roof biota that may influence the functionality and longevity of green roofs. Analogous to soil, the roof substrate likely contains a diverse array of microorganisms that help sustain the roof vegetation. While the physicochemical composition of green roof media has received considerable attention [14], fungal diversity and function on green roofs have not yet been examined despite their integral function in nutrient cycling, symbiosis, and plant productivity. The assemblage of microbes in roof growing media likely depends on numerous biotic and abiotic factors such as the plant community, initial substrate inoculum, local climate, moisture availability, and airborne inoculum [15], [16]. Bacteria and fungi are ubiquitous in ecosystems and due to their small sizes, many can readily disperse through the air [17]–[19]. As such, the distance that green roofs are from larger, intact vegetation patches such as city parks and forest fragments may be important in determining the numbers and types of microbial taxa able to disperse to a given roof. The initial fungal inoculum may also exhibit strong priority effects preventing new fungal species from establishing. Priority effects have been demonstrated for wood decomposer fungi [20], [21], ectomycorrhizal fungi [22], and yeasts [23], so it is plausible that historical contingencies from starting inoculum strongly influence green roof fungal composition.

This study aimed to address the following questions related to fungal communities in green roof substrates and nearby city park soils: 1) Do green roofs in New York City function as biodiversity reservoirs for fungi?; 2) Is there evidence for spatial structuring of green roof fungal communities across New York City? 3) Does vegetation type influence community composition of green roof fungi?; 4) How much overlap is there in fungal community composition of the green roof substrates and city park soils?

## Materials and Methods

### Study Site and Sample Collection

**Figure 1:**
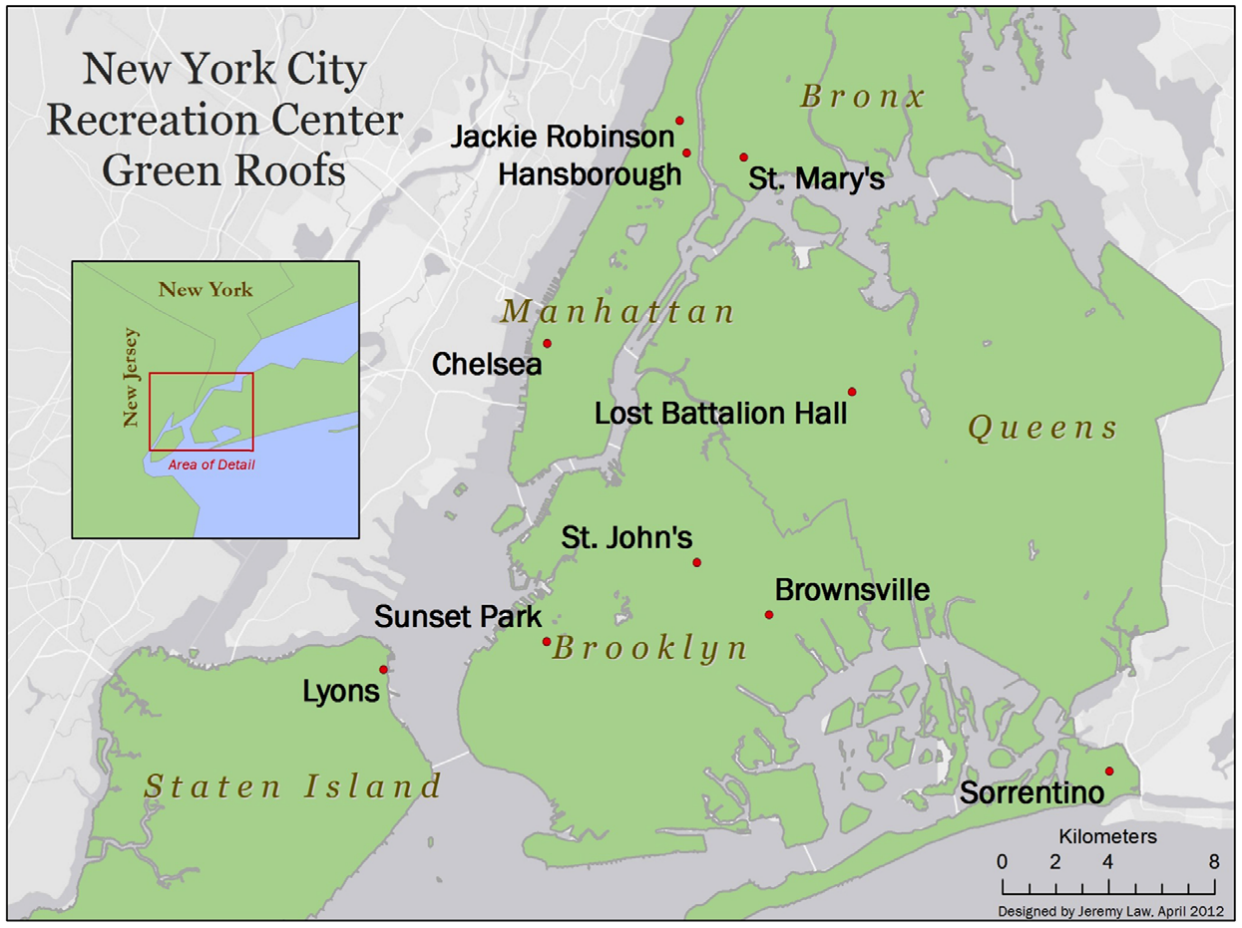
Locations of the ten green roofs sampled in this study, which were distributed across all five boroughs of New York City. The map was created by Jeremy Law at Columbia University.

This study was conducted on ten replicate green roofs and five city parks spanning the five boroughs of New York City (Fig. 1). All green roofs and parks are between 40.6–40.8°N, 73.7–74.0°W and 5–50 meters above sea level. Average annual precipitation for New York is approximately 1100 mm with a mean annual temperature of 16.4**°**C (Graphical Climatology 2012). All samples for the current study were taken in July 13–18, 2011.

The experimental green roofs were located on ten different recreation centers owned by the New York City Department of Parks and Recreation (Fig. 1). Each green roof had 12 planting boxes with each box having a dimension of 4 m by 2 m. Of the 12 boxes, six were installed with 10 cm of a commercially green roof growth media and the other six were installed with 15 cm of media. For this study, we only sampled planting boxes 15 cm of substrate. The boxes were divided into halves, with each half containing a subset of representative species from two native plant communities found in the New York City region: Hempstead Plains and Rocky Summit Grasslands (Table 1). The Hempstead Plains is a threatened native prairie community originally covering more than 24,000 ha on Long Island [24]. Nearly 200 species from 50 families comprise this vegetation type, with Poaceae and Asteraceae being the dominant plant families. Due to settlement and invasive species, <1% of the native vegetation remains [24]. Rocky Summit grasslands occur on the tops of mountains, ridges, and outcrops throughout Lower New England and the Hudson Highlands of New York state [25]. The Hempstead Plains and Rocky Summit plant communities were chosen because they support plant species that can tolerate environmental conditions typical of green roofs, such as limited water storage, thin soils, prolonged UV radiation, and high winds. From each of the native plant communities, eight representative species were selected for the experimental green roofs (Table 1). Plants were grown from seed at the Greenbelt Native Plant Center (Staten Island, NY) in local soil that was amended with compost and steam-sterilized prior to planting. Thus, the starting inoculum for all green roof plants was uniform. The boxes were installed in the spring of 2010, and the plants were installed in September and October of 2010.

**Table 1:** Plant species from the two native plant communities used in the experimental green roofs for this study.

Hempstead Plains		
*Plant Name*	*Latin Name*	*Plant Family*
Butterfly-weed	*Asclepias tuberose*	Apocynaceae
Gray goldenrod	*Solidago nemoralis*	Asteraceae
Hyssop-leaved boneset	*Eupatorium hyssopifolium*	Asteraceae
Smooth blue aster	*Symphyotrichum laeve*	Asteraceae
Yellow wild indigo	*Baptisia tinctoria*	Fabaceae
Little bluestem	*Schizachyrium scoparium*	Poaceae
Switchgrass	*Panicum virgatum*	Poaceae
Indian grass	*Sorghastrum nutans*	Poaceae
**Rocky Summit Grasslands**		
***Plant Name***	***Latin Name***	***Plant Family***
Stiff aster	*Ionactis linariifolius*	Asteraceae
Blackeyed Susan	*Rudbeckia hirta*	Asteraceae
Licorice-goldenrod	*Solidago odora*	Asteraceae
Bush-clover	*Lespedeza capitata*	Fabaceae
Narrowleaf mountainmint	*Pycnanthemum tenuifolium*	Lamiaceae
Poverty-oat grass	*Danthonia spicata*	Poaceae
Common Hairgrass	*Deschampsia flexuosa*	Poaceae
Deertongue	*Dichanthelium clandestinum*	Poaceae

**Figure 2:**
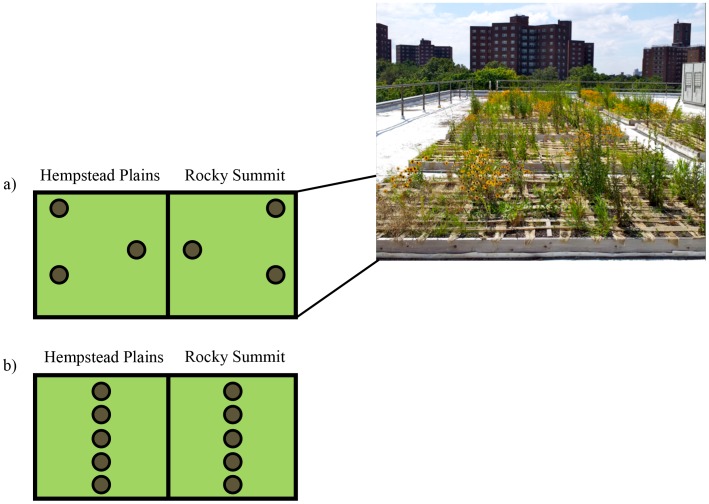
Sampling scheme for the general (a) and fine-scale (b) sampling with an image of a representative green roof. The general sampling scheme was used for all ten green roofs and the six cores were composited for three planting boxes on each roof. For the fine-scale sampling on the three target roofs, each core was treated as a separate sample.

To broadly survey fungal diversity found in the green roofs, we sampled substrate from planting boxes on all ten green roofs. We used 2.5 cm diameter soil corers to collect 10 cm-deep substrate samples from three roof boxes on each green roof and composited six substrate cores from each box (Fig. 2). For the broad sampling, soil cores from both vegetation types were composited, as we did not intend to use these samples to assess differences in fungal communities associated with the individual plant communities. Cores from each planting box were composited into sterile Whirl-Pak bags (Nasco, USA) and the soil corer was cleaned with EtOH between sample collections. To address our remaining three research questions, we conducted more intensive sampling on three of the green roofs located on the Lyons, Jackie Robinson, and Chelsea Recreation Centers. These roofs were chosen because they span the city geographically and have nearby ground-level parks. From three of the planting boxes on these roofs, 10 soil cores were separately collected: five from the Hempstead Plains plant community and five from the Rocky Summit plant community (Fig. 2). Since this sampling scheme was intensive in terms of the amount of substrate collected, we chose to only focus on three roofs to minimize the disturbance.

To compare the microbial communities from green roofs to other vegetated parts of the city, we collected soils from ground-level parks adjacent to the three green roofs that were more intensively sampled. These parks were all comprised of lawns and sparsely planted trees. We also collected soils from the High Line and Central Park, as these parks are principal green spaces in New York City. To obtain a representative sample of these park soils, we established three randomly located 5 m×5 m plots in all parks but Central Park, in which we sampled from 15 plots (a mixture of lawns and forested areas), due to its disproportionately large size relative to the other city parks. Five soil samples (0–10 cm) were composited from each plot into one sample, which was used as the unit of replication for all downstream analyses. Together, these five parks were suitable choices for this study because they are well-established parks that cover a vast range of area and are therefore represent the green environment in New York City. The permit for sampling both roofs and parks was granted by the City of New York Parks & Recreation Natural Resources Group (c/o Kristy King). The field studies did not involve any interaction with vertebrates or endangered/protected species.

### Soil and Microbial Analyses

Soil and roof substrate samples were homogenized using UV-sterilized 2 mm sieves, then analyzed for pH using a 1∶2 water ratio and a glass electrode. A suite of macro and micronutrients were analyzed for each sample at the Auburn University Soil Testing Laboratory (Al, USA). Inductively coupled plasma atomic emission spectroscopy was used to evaluate soil cations and trace metals. Loss on ignition was used to quantify total C and N.

To quantify total microbial biomass and bacterial:fungal ratios in roof substrates and park soils, we extracted and quantified phospholipid fatty acids (PLFAs). All lipids were extracted from 4 g soil by adding a 2∶1:0.8 (v/v/v) single-phase mixture of methanol, chloroform and citrate buffer. These lipids were then separated into neutral, glycolipid and phospholipid fractions with silica solid phase extraction columns [26]. We transesterified each phospholipid fraction into fatty acid methyl esters (FAMEs) using a 0.2 M solution of KOH and CH_3_OH. FAMEs were quantified with mass spectrometry using an Agilent 6980N capillary gas chromatography system in conjunction with the ChemStation software package (Agilent Technologies, Santa Clara, CA, USA), and identified using known fungal and bacterial fatty acid standards (Matreya LLC, Pleasant Gap, PA, USA). Total PLFAs were used to estimate total microbial biomass. Mole percentages of bacterial biomarkers (10Me16∶0, 10Me17∶0, 11∶0, 12∶0,14∶0,15∶0,16∶0,16∶1,9, 16∶1w6c, 16∶1w7t, 17∶0, 17∶0 delta, 18∶0, 18∶1w7c, 18∶1w7c/9t, delta 20∶0, 2-OH 10∶0, 2-OH 12∶0, 2-OH 14∶0, 2-OH 16∶0, 3-OH 12∶0, 3-OH 14∶0, a15∶0, i14∶0, i15∶0, i16∶0, i17∶0, i17∶1w8c) and fungal biomarker 18∶2w6,9 were used to quantify the relative biomass of bacteria and fungi [27].

### Illumina Sequencing

We used a barcoded high-throughput sequencing approach similar to that described in Caporaso et al. [28] to survey the diversity and composition of the fungal communities found in each of the collected soil and media samples. Briefly, DNA was extracted using a MoBio PowerSoil extraction kit following Lauber et al. [29] and the first internal transcribed spacer region (ITS1) of the fungal rRNA gene was amplified using the ITS1-F (CTTGGTCATTTAGAGGAAGTAA) and ITS2 (GCTGCGTTCTTCATCGATGC) primer pair [30], [31]. Both the forward and reverse primers also had the appropriate Illumina adapters, primer pad, and 2-bp linker sequences with the reverse primer containing a 12-bp error-correcting barcode unique to each sample. All DNA samples were amplified in triplicate in PCR reactions containing 13 µL water, 10 µL 5 Prime Hot Master Mix, 0.5 µL each of the forward and reverse primers (10 µM final concentration), and 1.0 µL genomic DNA. Reactions were held at 94°C for 3 min, with amplification proceeding for 35 cycles at 94°C for 45 s, 50°C for 60 s, and 72°C for 90 s; a final extension of 10 min at 72°C. The products of the triplicate PCR reactions were pooled, visualized on an agarose gel, and amplicon concentrations were quantified using the PicoGreen dsDNA assay. Amplicons from all samples were composited together in equimolar concentrations and sequenced using an Illumina HiSeq instrument at the University of Colorado, Boulder.

Reads were de-multiplexed, quality-filtered, and processed using the QIIME v. 1.5.0-dev pipeline [32]. Sequences were clustered into 97% similar operational taxonomic units (OTUs) using the UCLUST reference-based algorithm [33] with a manually curated ITS database composed of 97% clustered sequences retrieved from GenBank (http://www.ncbi.nlm.nih.gov/genbank/). High-quality sequences generated from each sample were rarified to 3200 sequences prior to downstream analyses. Taxonomy was assigned via the BLAST algorithm [34] with the aforementioned database.

### Statistical Analyses

Sequence data were analyzed at several different scales. At the broadest scale, we compared fungal community composition, substrate nutrient concentrations, and microbial biomass across all ten green roofs to examine the overall biogeographical structure. For the remaining statistical analyses, we only focused on the three target green roofs that were sampled more intensively. We evaluated whether or not fungal communities clustered by roof, by replicate box, and by plant community. We also compared fungal community composition of these three green roofs to fungal communities in the city park soils. We used the Bray-Curtis metric to calculate pairwise distances between fungal communities with the relative abundances of OTUs square root transformed prior to analysis. We used non-metric multidimensional scaling plots to visualize clustering patterns in the fungal communities with the statistical significance of the patterns determined using ANOSIM as implemented in PRIMER-E (v. 6). Differences in the relative abundance of specific taxa across roof locations were determined using multiple Kruskal-Wallis tests in R and applying false discovery rate corrections to p-values to account for the multiple comparisons. Tests were only performed for taxa with median relative abundances greater than 0.5% on any of the roof locations. Soil analyses, microbial biomass metrics, and the relative proportional abundance of OTUs within each phylum were compared between green roofs and parks using a multivariate ANOVA in SPSS (v. 20, Chicago, IL).

## Results

**Figure 3:**
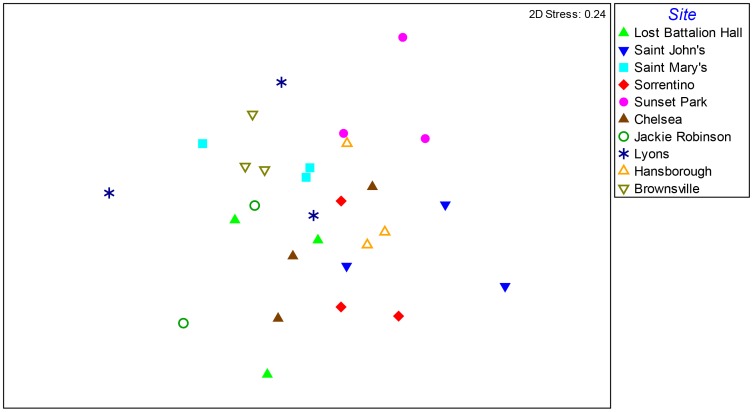
Non-metric multidimensional scaling plot of fungal communities across the ten green roofs. ANOSIM analysis revealed significant clustering of fungal communities across roofs.

Across the ten experimental green roofs, there was an average of 109 OTUs per roof. Taxonomic assignment of fungal OTUs revealed that the fungal community was dominated by the Ascomycota (59%) followed by the Glomeromycota (20%), Basidiomycota (13%), Zygomycota (6%), and Chytridiomycota (2%). The most abundant fungal orders were the Sordariales (17%), Pleosporales (16%), Microascales (14%), and the Glomerales (12%). The most abundant OTU in green roof substrates was classified as *Pseudallescheria fimeti* (Ascomycota), which represented 13.5% of all sequences in the roof samples. The next most abundant OTU in the roof substrates aligned to the genus *Glomus* (9.3% of sequences) followed by Ascomycota taxa in the genera *Lecythophora* (6.6%), *Peyronellaea* (6%), and *Thielavia* (4.6%). A total of 154 unique OTUs with 11,401 sequences aligned to taxa in the Glomeromycota phylum, which contains the mutualistic arbuscular mycorrhizal fungi. The Glomeromycota OTUs were classified as belonging to nine different genera (Table 2). The most diverse genus within the Glomeromycota was *Glomus* (82 OTUs) followed by *Rhizophagus* (36 OTUs) and *Acaulospora* (12 OTUs). In terms of sequence abundance, *Glomus* was also the most abundant genus, representing 49% of the total Glomeromycota sequences in the green roof samples. The fungal communities were not identical across the ten roofs, and there was a significant effect of roof location on fungal community composition (Fig. 3; R = 0.38, p = 0.001).

**Table 2:** Relative abundance of green roof OTUs aligning to fungal genera in the Glomeromycota (arbuscular mycorrhizal fungi).

Genus	OTU abundance	Sequence abundance
*Glomus*	0.53	0.62
*Rhizophagus*	0.23	0.17
*Acaulospora*	0.08	0.07
*Claroideoglomus*	0.05	0.01
*Paraglomus*	0.05	0.11
*Funneliformis*	0.03	0.01
*Entrophospora*	0.02	0.01
*Ambispora*	0.01	0.00
*Archaeospora*	0.01	0.01

**Figure 4:**
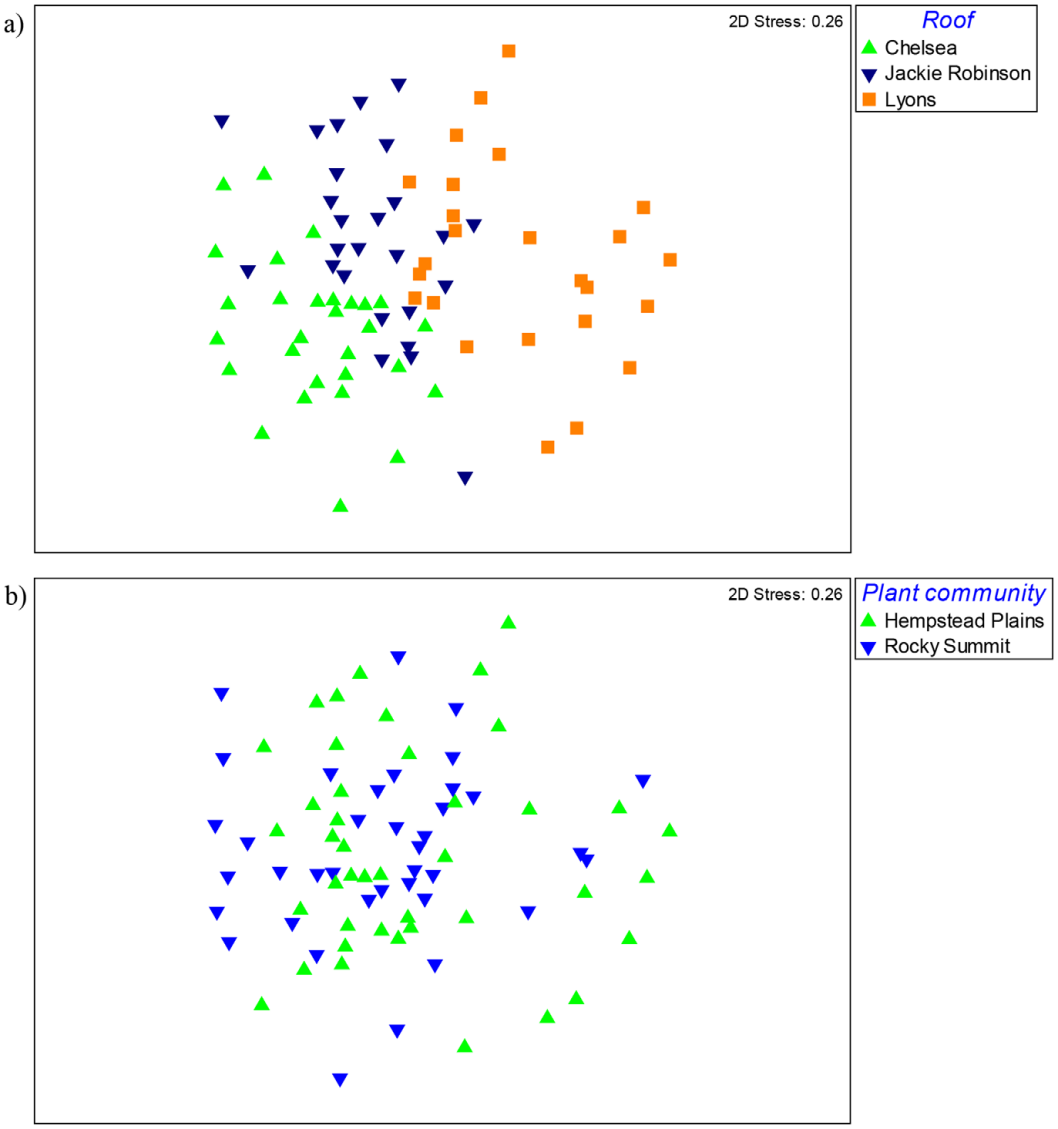
Non-metric multidimensional scaling plots of fungal communities for the three green roofs that were more intensively sampled. Fungal communities were significantly clustered by roof (a), but not by plant community (b).

**Figure 5:**
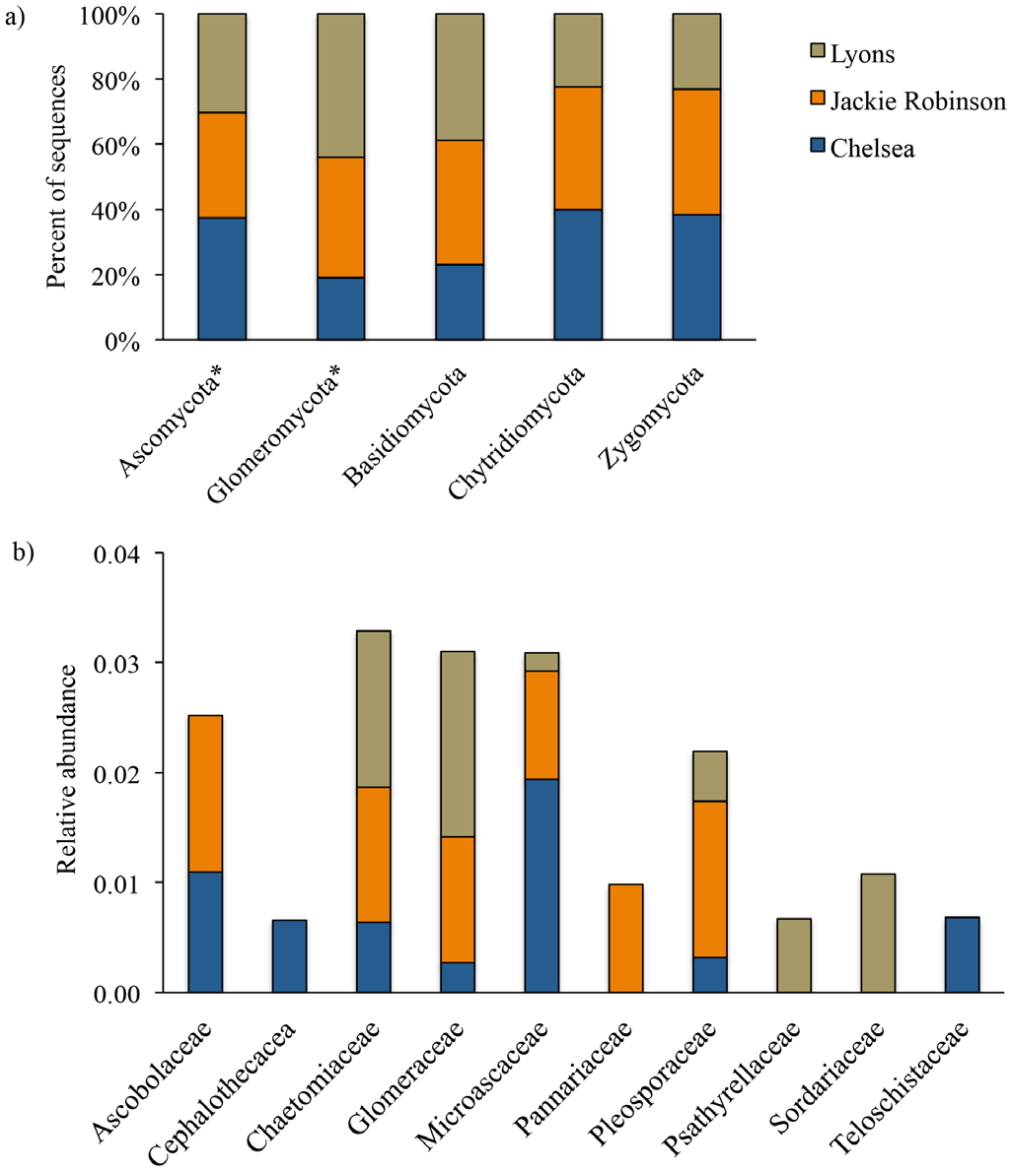
Proportional abundance of the fungal phyla (a) and families (b) that were responsible for the separation of fungal communities across the three intensively sampled green roofs. Fungal phyla (a) that had a non-random distribution across the three roofs are marked with an asterisk. All fungal families in panel b had non-random distributions across the three roofs.

The more intensive fine-scale sampling of the three target roofs also showed significant clustering of fungal communities by roof location (Fig. 4a; R = 0.35, p = 0.001). However, there were no differences in fungal communities across the two different native plant communities (Fig. 4b; R = 0.02, p = 0.08). Within a roof, fungal communities were significantly clustered by box (R = 0.7, p = 0.01). The clustering of fungal communities by roof was driven by differences in the relative abundance of fungal taxa from the Ascomycota (p<0.001) and the Glomeromycota (Fig. 5a; p<0.001). Specifically, the relative abundance of 10 fungal families was significantly different across the three roofs (Fig. 5b; p<0.05).

**Figure 6:**
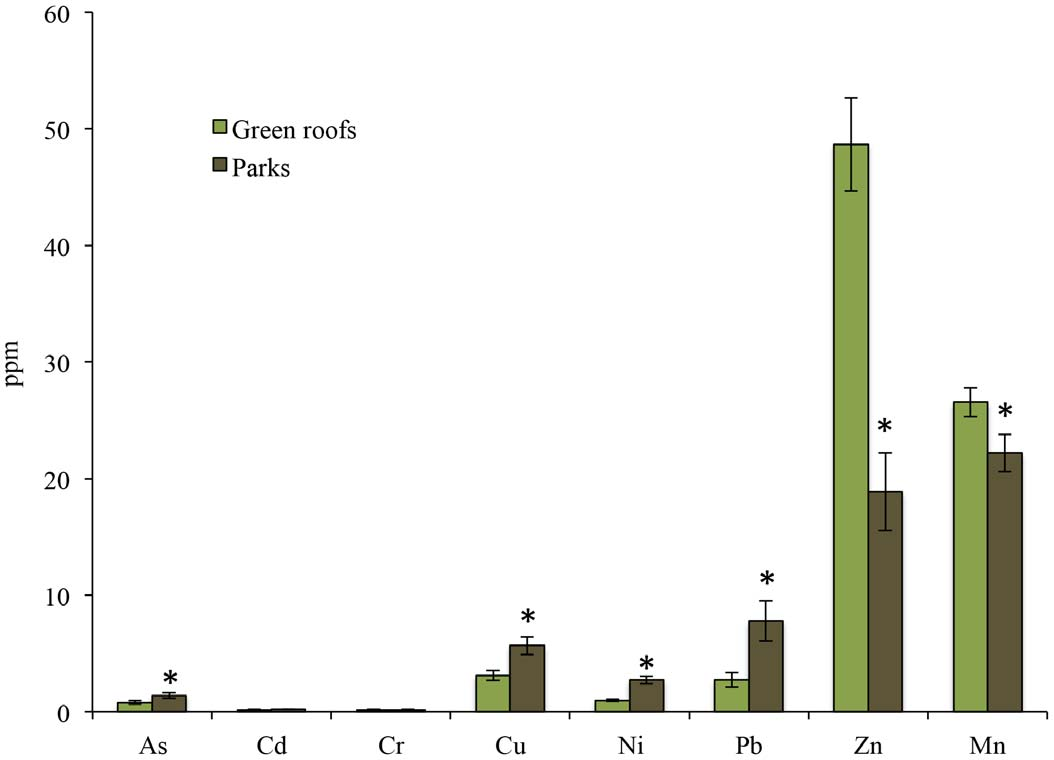
Concentrations (in ppm) of heavy metals from green roof substrates and park soils. Asterisks above the bars denote significant differences at p<0.05.

Green roof substrates and park soils differed in a number of physicochemical parameters (Table 3), including concentrations of heavy metals (Fig. 6). Park soils had significantly greater quantities of Al, total bases (Ca, Mg, and K), As, Cu, Ni, and Pb (p<0.05 for all contrasts). Green roof substrates, by contrast, had significantly higher quantities of Zn, Fe, Mn, Ba, and P compared to park soils. There were no notable differences in quantities of Cd, Cr, B, Mo, Na, or C to N ratios. Park soils also had significantly lower pH (6.6) than green roof substrates (7.3; p = 0.001). Total microbial biomass was significantly higher in park soils (99.3 nmol g^−1^ soil) relative to green roof substrates (78.7 nmol g^−1^ soil; F(1,56) = 6.2, p = 0.02). Bacterial to fungal ratios were also significantly higher in park soils (18.9) relative to green roof substrates (11.5; F(1,55) = 46.2, p<0.001).

**Table 3:** Data for soil and substrate nutrient analyses given as the mean (±SE).

	Green roofs	Parks
P*	367.5 (15.4)	92.3 (13.4)
Al*	177.1 (14.2)	266.7 (25.1)
B	1.6 (0.1)	0.7 (0.1)
Ba*	6.5 (0.1)	2.9 (0.4)
Fe*	111.9 (15.8)	58.9 (8.8)
Na	65.4 (7.1)	422.2 (242.5)
Total bases* (Ca+K+Mg)	4441.1 (154.3)	2594.7 (139.8)
C to N	13.6 (0.5)	14.7 (0.6)

Asterisks denote significant differences between green roof substrates and park soils at p<0.05. All nutrients are given in ppm except for C to N ratios.

**Figure 7:**
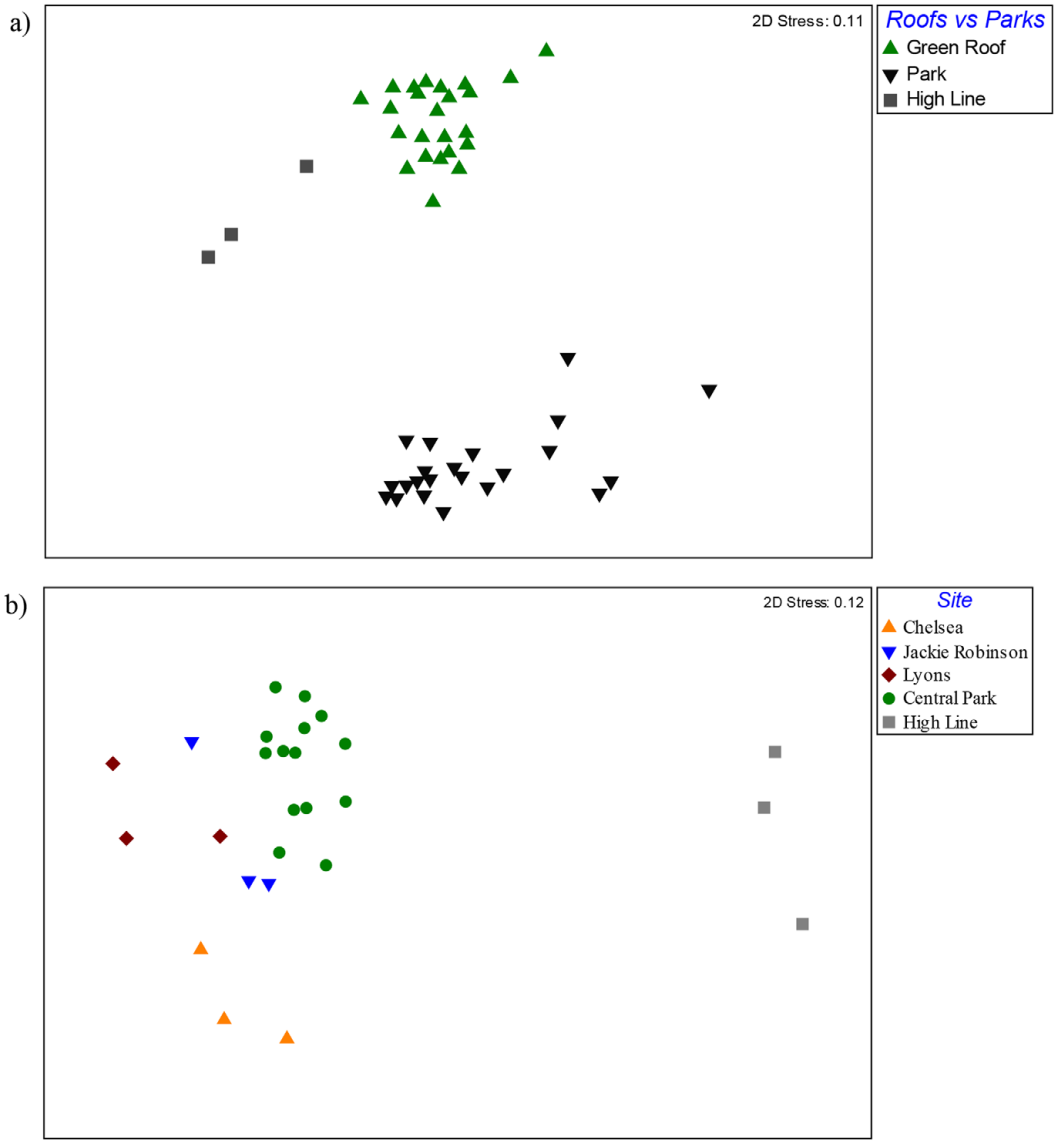
Non-metric multidimensional scaling plots of fungal communities sampled from green roofs and parks. Green roof fungal communities were distinct from city park soil communities with the High Line samples having a distinct composition from both roofs and other parks (a). City park fungal communities were also significantly clustered by site (b).

Fungal richness was higher in the parks than in green roofs, with an average of 154.3 OTUs found in park soils compared to an average of 109.1 OTUs in green roof substrates (F(1, 13.6) = 433.7; p<0.001). Fungal communities in green roof substrates were distinct from fungal communities in park soils (Fig. 7a; R = 0.86, p<0.001). There were 409 shared OTUs between green roof and park samples, which represented 54% of the green roof fungal OTUs and 33% of the park OTUs. Fungal community composition was significantly clustered for each city park (Fig. 7b; R = 0.77, p = 0.001). The High Line Park fungal community was dramatically different than the other city parks, and appeared to have a composition that was intermediate between parks and roofs (Fig. 7a,b). Central Park, which is the largest and most well known green space in New York City, had an average of 163 OTUs per sample and was dominated by OTUs aligning to the Eurotiales, Hypocreales, Pleosporales, Coniochaetales, Pezizales, Agaricales, and Glomerales (Table 4).

**Table 4:** The most abundant fungal OTUs from the Central Park samples.

Phylum	Order	Family	Genus	Total sequences
Ascomycota	Eurotiales	Trichocomaceae	*Paecilomyces*	3998
Ascomycota	Hypocreales	Bionectriaceae	*Myrothecium*	3988
Ascomycota	Hypocreales	Nectriaceae	*Fusarium*	2432
Ascomycota	Pleosporales	Pleosporaceae	*Curvularia*	2379
Ascomycota	Pleosporales	Cucurbitariaceae	*Pyrenochaetopsis*	1895
Zygomycota	Mortierellales	Mortierellaceae	*Mortierella*	1565
Ascomycota	Coniochaetales	Coniochaetaceae	*Lecythophora*	1522
Ascomycota	Pleosporales	Montagnulaceae	*Paraconiothyrium*	1434
Ascomycota	Pleosporales	Phaeosphaeriaceae	*Phaeosphaeriopsis*	890
Ascomycota	Pezizales	Tuberaceae	*Tuber*	864
Basidiomycota	Agaricales	Strophariaceae	*Hymenogaster*	722
Ascomycota	Glomerales	Plectosphaerellaceae	*Gibellulopsis*	686
Ascomycota	Hypocreales	Clavicipitaceae	*Metarhizium*	673
Ascomycota	Capnodiales	Davidiellaceae	*Cladosporium*	652
Ascomycota	Pleosporales	Didymellaceae	*Didymella*	581
Ascomycota	Pezizales	Tuberaceae	*Tuber*	557
Basidiomycota	Tremellales	Incertae sedis	*Trichosporon*	549
Zygomycota	Mortierellales	Mortierellaceae	*Mortierella*	547

Taxonomy is provided for the best match in Genbank. Only OTUs with sequences greater than 500 are reported.

**Figure 8:**
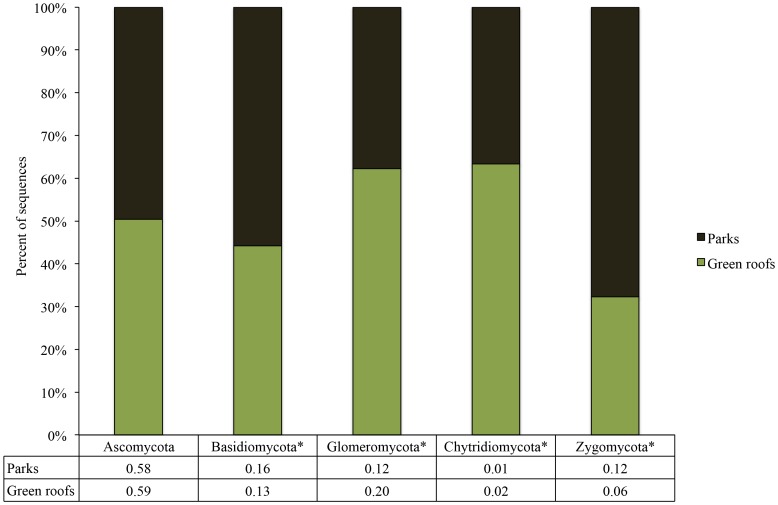
The relative abundance of fungal phyla detected from green roof substrates and city park soils. Asterisks denote significant differences at p<0.05. Numerical values for the proportional abundances of each fungal phylum in the parks compared to the green roofs are displayed below each bar.

Taxonomic assignment of OTUs revealed that across all green roof and city park samples there were significantly more Ascomycota fungi than Basidiomycota fungi, and that Ascomycota to Basidiomycota ratios were significantly higher on green roofs (5.3) compared to park soils (3.9; F(1,13.6) = 246.9; p<0.001; Fig. 8). The relative abundance of Glomeromycota was significantly greater in green roof samples compared to park samples (Fig. 8; F(1, 48) = 158; p<0.001). The relative abundance of Chytridiomycota was also significantly higher in the green roof samples compared to the park soils (Fig. 8; F(1,48) = 5.7; p = 0.02).

**Figure 9:**
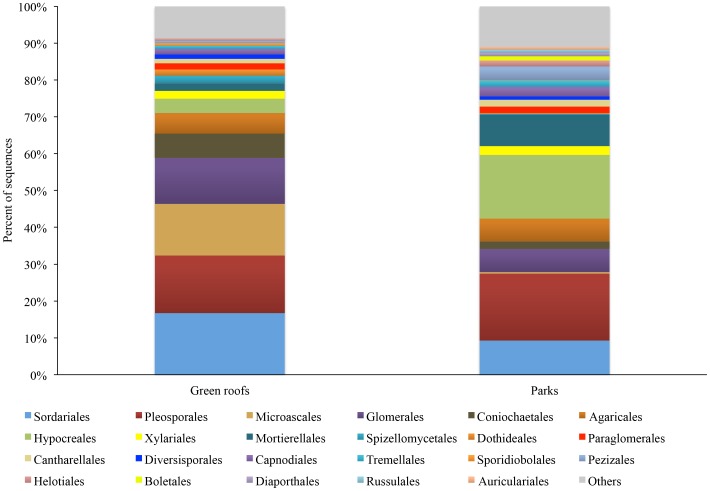
The relative abundance of the most dominant fungal orders detected from green roof substrates and city park soils.

Park soils had a lower abundance of Glomeromycota taxa compared to green roof media (p<0.05). As in the green roof samples, *Glomus* was the most abundant genus, containing 39% of all the Glomeromycota sequences. The most abundant Glomeromycota species, to which numerous OTUs aligned, was *Rhizophagus irregularis* (syn. *Glomus irregulare*), which accounted for 38% of all Glomeromycota sequences in green roof substrates. The relative abundance of fungal orders was also distinct across green roof and park soil samples, although many of the orders occurred in both park and green roof samples (Fig. 9).

## Discussion

One of the many recognized benefits of parks in urban environments is that they provide reservoirs of local biodiversity, and our data demonstrate that green roofs can serve a similar ecological function for soil fungi. We detected a surprisingly diverse assemblage of fungi from the green roof substrates, despite the extreme edaphic conditions of the green roof environment. The shallow growing media, physicochemical characteristics of the media, extreme fluctuations in water availability, and prolonged exposure to solar radiation may select for unique microbial taxa that are able to withstand these harsh conditions, and these factors likely contributed to the distinct fungal composition we observed in the green roof substrates relative to the ground-level city park soils. As one of the few studies to have investigated microbial diversity in New York City and the first to assess green roof microbial communities, our findings of a diverse microbial community spanning all the major fungal phyla demonstrate that even the small, vegetated areas of green roofs can maintain considerable fungal diversity. Since fungi are key components of ecosystems, understanding the factors that influence their diversity and distribution in human-dominated environments is crucial for optimizing the health and function of urban green spaces and their associated biotic communities.

The most abundant fungal taxa from the green roof substrates were closely related to taxa that are known to reside in disturbed environments and have been found to be resistant to some of the major contaminants in urban soils. For example, the most abundant OTU across all ten green roofs aligned to the species *Pseudallescheria fimeti*, which is a saprotrophic microfungus that has been found to be abundant in human-dominated environments in Europe [35]. *Pseudallescheria* was also detected in the park soils, although the sequence abundance was much lower. Interestingly, *Pseudallescheria* species have been found in other studies from soils that are amended with compost, such as agricultural sites, and in soils that are heavily polluted with hydrocarbons [36]. The next most abundant OTU in the green roof samples aligned to the genus *Peyronellaea*, which is an endophytic fungus that has been isolated from sites in China contaminated with heavy metals such as Pb and Zn [37]. Several other abundant OTUs aligned to the genera *Thielavia*, *Penicillium* and *Aspergillus*, which also have degradative capabilities in soils contaminated with pollutants. While concentrations of the heavy metals analyzed in green roof substrates and park soils were well below the levels typically found in contaminated soils, deposition rates of heavy metals and other pollutants have been steadily increasing in recent years. Since these potentially toxic elements can leach into water sources and cause harm to humans by inhalation [38], documenting microbial diversity and function in these environments is paramount for potential bioremediation efforts [39]. Further research is needed to determine if the various roof-associated microbes are simply resistant to urban pollutants or if they are actively involved in degradation and bioaccumulation.

The high richness and abundance of the Glomeromycota (AM fungi) suggests that green roof plants maintain their symbiotic fungal associations, even in the extreme environment of an urban rooftop. The top three most abundant OTUs all aligned to *Rhizophagus irregularis*, which is a widespread fungus known to associate with a variety of herbaceous plants. AM colonization levels of the plants were not quantified, but would be an important next step, as the most abundant AM taxa in the growing media may not correspond to the most abundant AM fungi on the plant roots. Additionally, AM fungi have recently undergone significant taxonomic rearrangement [40], so the true composition of the AM community would need to be determined from longer sequence reads that include more conserved genetic regions (e.g., 18S or 28S) for phylogenetic placement.

The distinct clustering of green roof from park fungal communities was likely influenced by differences in the physicochemical properties of the green roof growing media compared to the park soils. The green roof media is mostly comprised of inorganic expanded shale and compost, and as a result has low bulk density and higher concentrations of available nutrients compared to the park soils. The park plant communities were also dominated by turf grass, which may have further influenced their similarity to each other. At the landscape level, dispersal limitation of fungal spores may be an additional factor causing differences in roof and park fungal communities. Since the roofs were planted within a year of soil sampling, there may have not been sufficient time for fungal spores from either environment to overcome dispersal barriers. If dispersal limitation is an important driver of community composition, we may expect that with increasing time the fungal communities of city parks and their adjacent green roofs will converge, as stochastic events (e.g. strong wind currents and storms) allow spores to be transferred between the environments. Alternatively, the wide range of environmental differences between the green roofs and parks may result in environmental filtering overriding any successful colonization events, thereby limiting the establishment of new fungal species in the green roof environment [41]–[45]. Future studies that include sequencing of airborne fungi will be necessary to disentangle the roles of fungal dispersal limitation, priority effects, and environmental filtering [46].

While green roof fungal communities were distinct from park soil communities, when compared with each other, each roof had a clearly differentiated fungal assemblage. Since the growing media and plant communities were identical for all roofs, local-scale edaphic variations must have influenced the rapid divergence of these fungal communities after one year of planting. Additionally, local wind patterns and proximity to parks may have shaped the community of fungi dispersing into the green roofs. Rapid responses of microbial communities to new environments have been detected in other systems, and a recent study found that microbial community shifts correlated with decreased stress of plants adapting to novel environments [47]. Analogous microbial community shifts may be occurring within the first few years of green roof establishment, as plants are adapting to the new environment. Long-term monitoring of community composition will be necessary to gauge the response rate of microbial communities and the corresponding survival of plants in the novel environment.

Within a roof, fungal communities clustered by roof box, suggesting that there is some degree of spatial autocorrelation within the growing media. However, contrary to expectations, we did not find significant clustering of substrate microbes associated with the Hempstead Plains and Rocky Summit plant communities. Plant-soil feedbacks contribute to the heterogeneity of soil microbial communities in natural ecosystems, as individual plant species can create microsites that contain unique plant-associated microbial communities [48]. As such, we were expecting to find distinct microbial communities across the two native plant types with our fine-scale sampling. This lack of plant community effect may have been due to the recent time since planting (approximately one year), but it is also possible that Rocky Summit and Hempstead Plains plants are similar enough in their plant chemical constituents that microbial communities will not strongly differentiate between them.

We did not evaluate the spatial structure of microbes in the park soils, which is likely much more heterogeneous than the green roof microbial communities due to the higher diversity of plant species and growth forms. However, even with our small sample sizes from parks, we still found significant clustering of microbial communities within each park and distinct communities across all five park sites. Interestingly, soil fungi from the High Line Park had a fungal community that was intermediate in composition between that of the green roofs and the other city parks. This result may be due to several biotic and abiotic factors that the High Line Park shares with both green roofs and ground-level city parks. For example, the High Line is planted with a mixture of native plants (similar to green roofs), exotic grasses, and herbaceous species. However, it also contains woody species and succulents, similar to ground-level parks, and has an intermediate soil substrate depth of 45–90 cm. Additionally, the High Line is located at an elevation that is between city parks (ground level) and green roofs, which may influence the amount of sun and wind exposure that the site receives. The similarity of the Central Park fungal community composition to the Chelsea, Lyons, and Jackie Robinson parks may be due to the extensive area of lawn across all of the parks. Central Park, at 341 ha in size, is very heterogeneous in vegetation, but approximately one-third of the area is made up of turf grass. Since Central Park is one of the oldest and largest green spaces in New York City, the soil fungal communities of Central Park may be an important inoculum source for green roof fungal communities. However, other extensive parks in the outer Burroughs such as Van Cortland Park were not included in this study, and are probably more important for maintaining reservoirs of soil fungi outside of Manhattan.

### Conclusion

The practical and economic benefits of green roofs such as reducing building heating and cooling costs and managing stormwater are inextricably linked to the collective biota of the vegetated roof system. Soil microbial communities are the fabric of any vegetated terrestrial environment, and supporting populations of fungi offers another dimension for valuation of green roofs in urban environments. As the first study of fungal communities on green roofs, this work provides the baseline for future studies evaluating their function in the removal of pollutants from air deposition and subsequently from stormwater runoff.

In addition to studying microbial communities to understand their practical benefits, green roofs are also vegetated islands that can be used as model systems to study ecological processes [5] such as community assembly and population dynamics of microbial communities. Our understanding of the biogeographical distributions and local factors that maintain microbial diversity is still nascent. Linking assembly processes to factors such as the spatial configuration of the landscape, the roof vegetation, microbial priority effects, and the local microsite variables will enhance our theoretical foundations and understanding of microbial dynamics.

## References

[1] Carter T, Jackson CR (2007) Vegetated roofs for stormwater management at multiple spatial scales. Landscape and Urban Planning 80: 84–94.

[2] Del Barrio EP (1998) Analysis of the green roofs cooling potential in buildings. Energy and Buildings 27: 179–193.

[3] Mentens J, Raes D, Hermy M (2006) Green roofs as a tool for solving the rainwater runoff problem in the urbanized 21st century? Landscape and Urban Planning 77: 217–226.

[4] Brenneisen S (2006) Space for urban wildlife: designing green roofs as habitats in Switzerland. Urban Habitats 4: 27–33.

[5] Oberndorfer E, Lundholm J, Bass B, Coffman RR, Doshi H, et al (2007) Green roofs as urban ecosystems: Ecological structures, functions, and services. Bioscience 57: 823–833.

[6] McDonald RI, Kareiva P, Formana RTT (2008) The implications of current and future urbanization for global protected areas and biodiversity conservation. Biological Conservation 141: 1695–1703.

[7] Ockinger E, Dannestam A, Smith HG (2009) The importance of fragmentation and habitat quality of urban grasslands for butterfly diversity. Landscape and Urban Planning 93: 31–37.

[8] Strohbach MW, Haase D, Kabisch N (2009) Birds and the City: Urban Biodiversity, Land Use, and Socioeconomics. Ecology and Society 14.

[9] Monterusso MA, Rowe DB, Rugh CL (2005) Establishment and persistence of Sedum spp. and native taxa for green roof applications. Hortscience 40: 391–396.

[10] Villarreal EL, Bengtsson L (2005) Response of a Sedum green-roof to individual rain events. Ecological Engineering 25: 1–7.

[11] Lundholm J, MacIvor JS, MacDougall Z, Ranalli M (2010) Plant Species and Functional Group Combinations Affect Green Roof Ecosystem Functions. Plos One 5. 10.1371/journal.pone.0009677 PMC2837352 20300196

[12] VanWoert ND, Rowe DB, Andresen JA, Rugh CL, Fernandez RT, et al (2005) Green roof stormwater retention: Effects of roof surface, slope, and media depth. Journal of Environmental Quality 34: 1036–1044. 15888889 10.2134/jeq2004.0364

[13] Cook-Patton SC, Bauerle TL (2012) Potential benefits of plant diversity on vegetated roofs: a literature review. 106: 85–92. 10.1016/j.jenvman.2012.04.003 22575204

[14] Nagase A, Dunnett N (2011) The relationship between percentage of organic matter in substrate and plant growth in extensive green roofs. Landscape and Urban Planning 103: 230–236.

[15] Lennon JT, Aanderud ZT, Lehmkuhl BK (2012) Schoolmaster DR (2012) Mapping the niche space of soil microorganisms using taxonomy and traits. 93: 1867–1879. 10.1890/11-1745.1 22928415

[16] Fierer N, Lennon JT (2011) The generation and maintenance of diversity in microbial communities. 98: 439–448. 10.3732/ajb.1000498 21613137

[17] Green JL, Holmes AJ, Westoby M, Oliver I, Briscoe D, et al (2004) Spatial scaling of microbial eukaryote diversity. Nature 432: 747–750. 15592411 10.1038/nature03034

[18] Hallenberg N, Kuffer N (2001) Long-distance spore dispersal in wood-inhabiting Basidiomycetes. Nordic Journal of Botany 21: 431–436.

[19] Bowers RM, Sullivan AP, Costello EK, Collett JL, Knight R, et al (2011) Sources of Bacteria in Outdoor Air across Cities in the Midwestern United States. Applied and Environmental Microbiology 77: 6350–6356. 21803902 10.1128/AEM.05498-11 PMC3187178

[20] Dickie IA, Fukami T, Wilkie JP, Allen RB, Buchanan PK (2012) Do assembly history effects attenuate from species to ecosystem properties? A field test with wood-inhabiting fungi. Ecology Letters 15: 133–141. 22188588 10.1111/j.1461-0248.2011.01722.x

[21] Fukami T, Dickie IA, Wilkie JP, Paulus BC, Park D, et al (2010) Assembly history dictates ecosystem functioning: evidence from wood decomposer communities. Ecology Letters 13: 675–684. 20412280 10.1111/j.1461-0248.2010.01465.x

[22] Kennedy PG, Peay KG, Bruns TD (2009) Root tip competition among ectomycorrhizal fungi: Are priority effects a rule or an exception? Ecology 90: 2098–2107. 19739372 10.1890/08-1291.1

[23] Peay KG, Belisle M, Fukami T (2012) Phylogenetic relatedness predicts priority effects in nectar yeast communities. Proceedings of the Royal Society B-Biological Sciences 279: 749–758. 10.1098/rspb.2011.1230 PMC3248732 21775330

[24] Stalter R, Lamont EE (1987) Vegetation of Hempstead Plains, Mitchell Field, Long Island, New York. Bulletin of the Torrey Botanical Club 114: 330–335.

[25] Reschke C (1990) Ecological communities of New York State; Program NYNH, editor. Latham, NY: New York State Department of Environmental Conservation. 96 p.

[26] Frostegard A, Tunlid A, Baath E (1991) Microbial biomass measured as total lipid phosphate in soils of different organic content. Journal of Microbiological Methods 14: 151–163.

[27] Frostegard A, Baath E (1996) The use of phospholipid fatty acid analysis to estimate bacterial and fungal biomass in soil. Biology and Fertility of Soils 22: 59–65.

[28] Caporaso JG, Lauber CL, Walters WA, Berg-Lyons D, Huntley J, et al (2012) Ultra-high-throughput microbial community analysis on the Illumina HiSeq and MiSeq platforms. Isme Journal 6: 1621–1624. 22402401 10.1038/ismej.2012.8 PMC3400413

[29] Lauber CL, Hamady M, Knight R, Fierer N (2009) Pyrosequencing-Based Assessment of Soil pH as a Predictor of Soil Bacterial Community Structure at the Continental Scale. Applied and Environmental Microbiology 75: 5111–5120. 19502440 10.1128/AEM.00335-09 PMC2725504

[30] Gardes M, Bruns TD (1993) ITS primers with enhanced specificity for basidiomycetes - application to the identification of mycorrhizae and rusts. Molecular Ecology 2: 113–118. 8180733 10.1111/j.1365-294x.1993.tb00005.x

[31] Bellemain E, Carlsen T, Brochmann C, Coissac E, Taberlet P, et al (2010) ITS as an environmental DNA barcode for fungi: an in silico approach reveals potential PCR biases. Bmc Microbiology 10: 9. 20618939 10.1186/1471-2180-10-189 PMC2909996

[32] Caporaso JG, Kuczynski J, Stombaugh J, Bittinger K, Bushman FD, et al (2010) QIIME allows analysis of high-throughput community sequencing data. Nature Methods 7: 335–336. 20383131 10.1038/nmeth.f.303 PMC3156573

[33] Edgar RC (2010) Search and clustering orders of magnitude faster than BLAST. Bioinformatics 26: 2460–2461. 20709691 10.1093/bioinformatics/btq461

[34] Altschul SF, Madden TL, Schaffer AA, Zhang JH, Zhang Z, et al (1997) Gapped BLAST and PSI-BLAST: a new generation of protein database search programs. Nucleic Acids Research 25: 3389–3402. 9254694 10.1093/nar/25.17.3389 PMC146917

[35] Kaltseis J, Rainer J, De Hoog GS (2009) Ecology of *Pseudallescheria* and *Scedosporium* species in human-dominated and natural environments and their distribution in clinical samples. Medical Mycology 47: 398–405. 19085459 10.1080/13693780802585317

[36] April TM, Abbott SP, Foght JM, Currah RS (1998) Degradation of hydrocarbons in crude oil by the ascomycete Pseudallescheria boydii (Microascaceae). Canadian Journal of Microbiology 44: 270–278. 9606909 10.1139/w97-152

[37] Li HY, Li DW, He CM, Zhou ZP, Mei T, et al (2012) Diversity and heavy metal tolerance of endophytic fungi from six dominant plant species in a Pb-Zn mine wasteland in China. Fungal Ecology 5: 309–315.

[38] Chillrud SN, Bopp RF, Simpson HJ, Ross JM, Shuster EL, et al (1999) Twentieth century atmospheric metal fluxes into Central Park Lake, New York City. Environmental Science & Technology 33: 657–662. 21850150 10.1021/es9807892 PMC3155985

[39] Gadd GM (2010) Metals, minerals and microbes: geomicrobiology and bioremediation. Microbiology-Sgm 156: 609–643. 10.1099/mic.0.037143-0 20019082

[40] Kruger M, Kruger C, Walker C, Stockinger H, Schussler A (2012) Phylogenetic reference data for systematics and phylotaxonomy of arbuscular mycorrhizal fungi from phylum to species level. New Phytologist 193: 970–984. 22150759 10.1111/j.1469-8137.2011.03962.x

[41] Baas-Becking L (1934) Geobiologie of Inleiding Tot de Milieukunde. The Hague: Van Stockkum & Zoon.

[42] Fierer N (2008) Microbial biogeography: patterns in microbial diversity across space and time. ASM Press. 95–115.

[43] De Deyn GB, Van der Putten WH (2005) Linking aboveground and belowground diversity. Trends in Ecology & Evolution 20: 625–633. 16701446 10.1016/j.tree.2005.08.009

[44] Beijerinck M (1913) De Infusies en de Ontdekking der Backter ien, Jaarboek van de Koninklijke Akademie v. Wetenschappen. (Muller, Amsterdam).

[45] Martiny JBH, Bohannan BJM, Brown JH, Colwell RK, Fuhrman JA, et al (2006) Microbial biogeography: putting microorganisms on the map. Nature Reviews Microbiology 4: 102–112. 16415926 10.1038/nrmicro1341

[46] Peay KG, Garbelotto M, Bruns TD (2010) Evidence of dispersal limitation in soil microorganisms: Isolation reduces species richness on mycorrhizal tree islands. Ecology 91: 3631–3640. 21302834 10.1890/09-2237.1

[47] Lau JA, Lennon JT (2012) Rapid responses of soil microorganisms improve plant fitness in novel environments. 109: 14058–14062. 10.1073/pnas.1202319109 PMC3435152 22891306

[48] Bever JD, Platt TG, Morton ER (2012) Microbial population and community dynamics on plant roots and their feedbacks on plant communities. Annual Review of Microbiology 66: 265–283. 10.1146/annurev-micro-092611-150107 PMC3525954 22726216

